# Interaction between the flagellum of *Candidatus* Liberibacter asiaticus and the vitellogenin-like protein of *Diaphorina citri* significantly influences *C*Las titer

**DOI:** 10.3389/fmicb.2023.1119619

**Published:** 2023-04-18

**Authors:** Tao Peng, Yingzhe Yuan, Aijun Huang, Jun He, Shimin Fu, Shuo Duan, Long Yi, Chenyang Yuan, Huizhu Yuan, Xuefeng Wang, Changyong Zhou

**Affiliations:** ^1^National Citrus Engineering Research Center, Citrus Research Institute, Southwest University, Chongqing, China; ^2^National Navel Orange Engineering Research Center, Gannan Normal University, Ganzhou, China; ^3^Key Laboratory of Entomology and Pest Control Engineering, College of Plant Protection, Southwest University, Chongqing, China; ^4^Institute of Plant Protection, Chinese Academy of Agricultural Sciences, Ministry of Agriculture and Rural Affairs, Beijing, China

**Keywords:** *C*Las, Huanglongbing, *Diaphorina citri*, flagella, vitellogenin

## Abstract

Huanglongbing (HLB) is a global devastating citrus disease that is mainly caused by “*Candidatus* Liberibacter asiaticus” (*C*Las). It is mostly transmitted by the insect Asian citrus psyllid (ACP, *Diaphorina citri*) in a persistent and proliferative manner. *C*Las traverses multiple barriers to complete an infection cycle and is likely involved in multiple interactions with *D. citri*. However, the protein–protein interactions between *C*Las and *D. citri* are largely unknown. Here, we report on a vitellogenin-like protein (Vg_VWD) in *D. citri* that interacts with a *C*Las flagellum (flaA) protein. We found that *Vg_VWD* was upregulated in *C*Las-infected *D. citri.* Silencing of *Vg_VWD* in *D. citri via* RNAi silencing significantly increased the *C*Las titer, suggesting that *Vg_VWD* plays an important role in the *C*Las*–D. citri* interaction. *Agrobacterium*-mediated transient expression assays indicated that Vg_VWD inhibits BAX- and INF1-triggered necrosis and suppresses the callose deposition induced by flaA in *Nicotiana benthamiana*. These findings provide new insights into the molecular interaction between *C*Las and *D. citri*.

## 1. Introduction

Citrus Huanglongbing is the most devastating citrus disease worldwide and is associated with the phloem-colonizing pathogenic α-proteobacterium “*Candidatus* Liberibacter spp.” Among the three known species “*Ca.* L. asiaticus” (*C*Las), “*Ca.* L. americanus” (*C*Lam), and “*Ca.* L. africanus” (*C*Laf) (Bové, 2006; Andrade et al., 2020), *C*Las is the most aggressive and is transmitted by the Asian citrus psyllid (ACP, *Diaphorina citri*) in a persistent-propagative manner (Andrade et al., 2020; Zhou, 2020). After being ingested by *D. citri* from the phloem sap of diseased citrus, *C*Las initially infects the intestinal epithelium and then crosses the basal lamina into the midgut visceral muscles, from where it spreads into the hemolymph, followed by the salivary glands, and then back into citrus with the *D. citri* feeding process (Wei and Li, 2016; Chen et al., 2022). The protein–protein interactions between the host and pathogen are extremely important for pathogen colonization, movement, and transmission. Studying the interaction mechanisms between *C*Las and *D. citri* is important for finding solutions for this disease. However, little has been reported on the abovementioned issue.

The bacterial flagellum is a complex organelle embedded within the cell envelope that has long extracellular helical filaments (flagellar filaments). The flagellum is critical for bacterial motility, niche colonization, and pathogenesis (Subramanian and Kearns, 2019). As a transboundary pathogen, *C*Las retains a complete family of 30 flagella-encoding genes on its significantly reduced genome, among which the flagellin (flaA, CLIBASIA_02090) gene encodes monomers of the filaments (Duan et al., 2009). *C*Las *flaA* encodes 452 amino acids and contains a conserved 22-amino acid domain (flg_22_) at positions 29 to 50 of the N terminus. It has pathogen-associated molecular pattern (PAMP) activity and induces plant innate immunity, including plant cell death and callose deposition (Zou et al., 2012). The expression pattern of *C*Las flagellar region genes varies in different hosts, with high expression in *C*Las-infected *D. citri* and low or no expression in susceptible *Citrus* plants (Yan et al., 2013; Andrade et al., 2020). No flagellar morphology was observed in *C*Las from citrus samples, and flagella-like structures in *C*Las were observed from the midgut of the *C*Las-infected *D. citri* (Andrade et al., 2020). *C*Las traverses multiple barriers in *D. citri* to complete the infection cycle, and the flagella enhance its motility to a favorable location for better colonization. Genomic and transcriptomic studies indicate that *D. citri* does not have an intact immune system like model insects such as *Drosophila* (Wulff et al., 2014; Vyas et al., 2015; Wang et al., 2017). Due to the lack of adaptive immunity and immunity pathways against Gram-negative bacteria, it is susceptible to infection by *C*Las, which can easily replicate and spread *in vivo* (Vyas et al., 2015; Arp et al., 2017).

Vitellogenin (Vg) belongs to the large lipid transfer protein (LLTP) superfamily (Sarkar and Ghanim, 2022). As a yolk precursor protein, Vg is present in almost all oviparous organisms, including insects, crustaceans, fishes, birds, amphibians, and reptiles (Zhang et al., 2011; Kruse et al., 2018). Vg was originally thought to be a female-specific protein. However, research has shown that Vg is not only involved in yolk protein formation but also plays a sex-independent role associated with immune function in non-mammalian vertebrates and invertebrates. In insects, Vg is generally synthesized in the fat body and secreted into the hemolymph and other tissues to perform functions. Vg usually serves as a pattern recognition molecule to recognize pathogens (Liu et al., 2009; Arrese and Soulages, 2010). Recent reports have shown that Vg can interact with PAMPs such as bacterial outer membrane proteins, flagella, and pili and acts as one of the pattern recognition receptors (PRRs) inducing host immunity (Li et al., 2008; Zhang et al., 2011; Li et al., 2017; Sarkar and Ghanim, 2022). Hemocyte-produced Vg interacts with rice stripe virus (RSV) and is positively correlated with RSV survival in *Laodelphax striatellus* (Huo et al., 2018), acts as an antioxidant for immunity in bees and *Caenorhabditis elegans* (Nakamura et al., 1999; Seehuus et al., 2006), and inhibits *Staphylococcus aureus* by binding to the lipoteichoic acids of the bacterial surface in *Homarus* (Hanada et al., 2011). Moreover, Vg also acts as a salivary protein involved in the insect–plant host interactions (Jaiswal et al., 2021). Vg generally contains three conserved domains: the lipoprotein amino-terminal region located at the N terminus, the DUF of unknown function, and the VWD domain at the C terminus. LPD_N is a very conserved and characteristic domain, DUF is a domain whose function is still unknown, and the VWD domain is rich in cysteines, which are associated with the formation of disulfide bonds (Qiao et al., 2021). The VWD domain has been found in the Vg of several vertebrates, crustaceans, and insects, and the domain induces the binding of oocyte membrane receptors to Vg (Opresko and Wiley, 1987; Wu et al., 2018; Faiz et al., 2019). In addition, VWDs are also present in several other proteins such as mucins and banded adhesion proteins, where the sphericity of this domain enhances the function of mucins and underlies the adhesion function in other proteins such as integrins and zonadhesins (Toribara et al., 1997; Qiao et al., 2021).

In this study, we screened the *D. citri* membrane protein library using *C*Las flagellin (flaA) as bait and found that flaA interacted with a vitellogenin-like protein (Vg_VWD Gene ID: LOC103523873), which was further confirmed by glutathione S-transferase (GST) pull-down and co-immunoprecipitation (Co-IP) assays. The transcription level of *Vg_VWD* was upregulated in *C*Las-infected *D. citri* and was highly expressed in the fat body and salivary glands. Silencing the expression of *Vg_VWD* significantly increased the *C*Las titer at different time points. Vg_VWD could suppress BAX- and INF1-triggered hypersensitive cell death and inhibit flaA-induced callose deposition in *Nicotiana benthamiana*.

## 2. Materials and methods

Overall, all the gene constructs were confirmed by Sanger sequencing. The primers used in this study are listed in Supplementary Table 1. The strains and plasmids used in this study are listed in Supplementary Table 2.

### 2.1. Insect rearing and plant growth

Uninfected *D. citri* used were reared in a greenhouse at the National Navel Orange Engineering Research Center, Gannan Normal University, Ganzhou, China. *C*Las-infected *D. citri* were collected near Tandong orchard (latitude 25°47′5″ north and longitude 114°52′4″ east). Infected *D. citri* and uninfected *D. citri* were reared separately in cages (60 cm × 60 cm × 90 cm). For this experiment, one to two pots of healthy citrus or *Murraya exotica* were maintained in the rearing cages under the conditions of 27 ± 1°C and RH of 70 ± 5%, with a 14-h light/10-h dark cycle. Wild-type (WT) *N. benthamiana* plants were grown in a growth chamber maintained at 25 ± 2°C and an RH of 70 ± 5%, with a 16-h light/8-h dark cycle.

### 2.2. Gene cloning and vector construction

The full length of *Vg_VWD* (Gene ID: LOC103523873, XP_008487105.1, 557 aa) was obtained by quantitative real-time (qRT)-PCR using total RNA isolated from *D. citri*, cloned into the pCE2 TA/Blunt-Zero vectors (Vazyme, China), and then sequenced. The ClonExpress II One Step Cloning Kit (Vazyme, China) was used to insert *Vg_VWD* into the prokaryotic expression vector (pGEX-4T-1, GST-tagged protein), yeast two-hybrid (Y2H) vector PPR3-N (prey), vector pgR107 of potato virus X (PVX), and pull-down vector PMAL-C2X (PBM-tagged protein). The sequence of the *C*Las flagellin (*flaA*) was derived from the whole-genome sequence of the strain Psy62 (taxid: 537021, GenBank accession no. CP001677) (Duan et al., 2009). It was cloned into the Y2H vectors pBT3-N, pBT3-STE, pDHB1 (bait plasmid), pull-down vector (pGEX-4T-1, GST-tagged protein), and PVX vector pgR107.

### 2.3. Yeast two-hybrid (Y2H) assay

The full *flaA* gene (Gene ID: CLIBASIA_02090, 1359 dp) was cloned in-frame into the vector (pBT3-N, pBT3-STE, and pDHB1) as the bait. The self-activation and functional validation of the bait plasmids referred to the methods of Than et al. (2016) and Liang et al. (2022). In brief, the NMY51 yeast cells containing the plasmid were transferred to DDO (SD/-His/−Leu with agar), TDO (SD/-His/−Leu/−Trp with agar), and QDO (SD/-His/−Leu/−Trp/−Ade with agar) for observation and recording of the growth. The certified bait plasmids were transformed into NMY51 yeast cells according to the manufacturer’s instructions to produce the yeast receptor cells containing the decoy plasmids. The *D. citri* membrane library plasmids were transferred into receptor cells, coated on QDO-medium, and incubated at 28°C for 3–5 days, following which the plasmids were extracted and sequenced from the grown single colonies. The obtained candidate proteins were verified by Y2H protein–protein, and the interaction intensity was verified by the *β*-galactosidase colorimetric reaction.

### 2.4. Protein expression and GST pull-down analysis

The full *flaA* (CLIBASIA_02090, 1,359 dp) was cloned into pGEX-4T-1 for fusion with the GST tag, and *Vg_VWD* was cloned into PMAL-C2X for fusion with the MBP tag. The recombinants were transformed into competent *Escherichia coli* Rosetta (DE3) cells for expression and purified with the GST Tag Protein Purification Kit (Beyotime Biotechnology, China) after IPTG (1 mM, 20°C for 8 h). For the experimental and control groups, 500 μg of GST and GST-flaA were added to fully bind the solutions with 50% glutathione-agarose resin at 4°C. The supernatant was removed by centrifugation, and 1 mL of PBST was added to wash off the unbound protein in the resin, following which 500 μg of the MBP/His-Vg_VWD protein was added and incubated overnight at 4°C. The products were rinsed three times with 1 mL of pre-cooled PBST, and RIPA-buffered cell lysate and loading buffer were added and mixed well, boiled for 5–10 min, and then the supernatant was collected by centrifugation. The supernatant was separated by SDS-PAGE and subjected to immunoblotting analysis.

### 2.5. *In vivo* Co-IP assay

The Co-IP assays were performed as previously described with a slight modification (Wang et al., 2016). In brief, His and Flag were tagged to the N terminus and C terminus of *Vg_VWD*, respectively, and HA was tagged to the N terminus of flaA. The construct fusion plasmids pFastBac1-Vg_VWD and pFastBac1-flaA were transfected into Sf9 cells to detect protein expression. The fusion plasmids were then co-transfected into Sf9 cells, and the expression of the co-transfected proteins was detected. The co-transfected cell lysate samples were collected for subsequent experiments. First, the co-transfected cell lysate was pretreated, the lysate was incubated with the protein A magnetic column to prevent non-specific binding between the lysate and the magnetic column, and the incubated flow-through was taken for Co-IP experiments. Second, the experimental and control groups were set up: the flag antibody or IgG antibody was hung onto the protein A magnetic column, then the supernatant was lysed after incubation with mixed magnetic beads (4°C, 3 h), followed by washing thoroughly with 1 mL of ice-cold PBS buffer for three times to elute the protein, which was collected for Western blot detection.

### 2.6. Collection of *Diaphorina citri* tissues

According to the morphological differentiation method for *D. citri* nymphs, 3–5 instar nymphs were prepared, with three biological replicates for each instar and 15 nymphs per replicate. For the collection of different gender adults, the 5th instar nymphs were selected and transferred to a single *M. exotica* seedling for feeding. Then, the adults were collected on days 3, 5, and 10 after emergence, respectively. Adults of different genders were distinguished under a microscope and were separately collected. There were three biological repeats and five male or female adults of *D. citri* per replicate.

The *C*Las-infected adults of *D. citri* were collected from orchards, where *C*Las-positive rates ranged between 80% and 95% (Supplementary Figure 2A). Six organs including the midgut, bacteriomes, testis, ovary, fat body, and hemolymph were dissected under the insect-dissecting microscope, and the hemolymph was collected according to Kruse et al.’s method (2018). Tissues collected from 30 *D. citri* adults (male:female = 1:1) were taken as one biological replicate, and three biological replicates were used in experiments. In addition, the salivary glands and Malpighian tubes were collected with three biological replicates each from 100 adults *D. citri* (male:female = 1:1). Dissected tissues were transferred to 1.5-mL centrifuge tubes (Trizol, 500 μL) using forceps. Subsequently, the total nucleic acids were extracted.

### 2.7. *C*Las titer quantification within *Diaphorina citri*

The DNA extraction of a single *D. citri via* method A was used to detect the *C*Las-infection rate of *D. citri* in the field and method B was used to detect the *C*Las titer in infected *D. citri*. Method A is performed according to the instructions for the Animal Tissue Direct PCR Kit as follows: (1) the *D. citri* were kept on ice for 3–5 min; (2) each *D. citri* was transferred *via* forceps to PCR tubes with premixed 12.5 μL buffer AL and 0.5 μL Foregene protease; (3) mashed with a small sterilized grinding rod homogenate, and the homogenate was treated under the conditions of 65°C for 20 min and 95°C for 5 min; and (4) centrifuged at 12,000 rpm for 5 min. The PCR detection system has been carried out according to Shen et al. (2022). Two μL of the supernatant obtained from method A was aspirated as the PCR template with primers OI1 and OI2 (Supplementary Table 1). Method B extracted DNA from *D. citri* according to the CTAB method, and the specific steps were performed according to Quintana et al. (2022). A two-step assessment of *C*Las titer in a single *D. citri* was addressed: (1) the presence of *C*Las was detected by ordinary PCR; and (2) the *C*Las titer in infected *D. citri* was quantified by qPCR.

To perform qPCR, the total DNA of a single *C*Las-infected *D. citri* was diluted to 100 ng/μL, and the copy number of *C*Las per 100 ng of total DNA was detected by absolute quantification. Probe qPCR experiments were performed with Premix (TakaRa, Dalian, China) using probe primers HLBr and HLB4G, and sequence information is provided in Supplementary Table 1. A mixture (20 μL) of Premix (10 μL), probe (0.3 μL), each primer (0.4 μL), and *D. citri* template DNA [1 μL (100 ng)] was reacted on a Light Cycler 96 SYBR Green I Master (Roche). The qPCR conditions were as follows: 3 min at 95°C; 40 thermal cycles (10 s at 95°C; 30 s at 60°C); and 30 s at 37°C. At least three technical replicates were performed for each sample. The equation for absolute quantification of *C*Las was *y* = −4.11x + 55.508 (*R*^2^ = 0.9964), where *y* is the Ct value, *x* is the copy number, and the *C*Las titer is 10x per 100 ng of the *D. citri* DNA.

### 2.8. RNA extraction and RT-qPCR analysis

The *D. citri* total RNA was extracted using Trizol regent (Invitrogen, Carlsbad, CA, United States) according to the manufacturer protocol. The synthesis of cDNA was performed by using a PrimeScript RT reagent kit with gDNA Eraser (TaKaRa, Dalian, China) with 1 μg of total RNA reverse transcribed for each age of *D. citri* and 0.5 μg of total RNA reverse transcribed for each tissue. The cDNA was diluted 3–5 times and then subjected to RT-qPCR, and a reaction system (20 μL) was applied, including 10 μL of 2 × TB Green Premix (TaKaRa, Dalian, China), 7 μL of DNase/RNase-free water, 0.8 μL of each primer (10 μM), 0.4 μL of ROX reference, and 1 μL of diluted cDNA template. The PCR cycling consisted of an initial activation step at 95°C for 3 min, followed by 40 cycles of 95°C for 5 s and 60°C for 34 s, and a single collection at 72°C for 30 s. The *DcGAPDH* gene was used as an internal control. The relative expression value was calculated with three biological and technical replicates, and the calculation was accordant with the 2^−ΔΔCT^ quantification method (Livak and Schmittgen, 2001). The primers are listed in Supplementary Table 1.

### 2.9. DsRNA synthesis and RNAi silencing

The primers specific (Supplementary Table 1) for *Vg_VWD* and *GFP* were designed to synthesize dsRNA by using the T7 High Yield Transcription Kit (Thermo Scientific, Wilmington, DE, United States) according to the manufacturer’s instructions. The *D. citri* adults addressed for dsRNA injection were *ca.* 95% of *C*Las infection rates (Supplementary Figure 2B). The dsRNAs of *Vg_VWD* and *GFP* were injected as experimental and control groups, respectively, and the injected *D. citri* were transferred to *M. exotica* seedlings for rearing (Supplementary Figure 3). The total DNA of *D. citri* was extracted and quantified to 100 ng/μL at 6, 12, and 24 h after injection. The titer of *C*Las per 100 ng total DNA of treated *D. citri* was determined. The experiments for each group were designed with at least three biological replicates (each with five males or females) and three technical replicates.

### 2.10. Agro-infiltration assay in *Nicotiana benthamiana*

The full-length sequences of *flaA* (1,359 dp) and *Vg_VWD* (1,674 dp) were inserted into the binary vector potato virus X (PVX) digested by *Cla*I/*Sal*I (Liu et al., 2019). PVX-GFP was used as a negative control and PVX-BAX and PVX-NIF1 as positive controls. The plasmids PVX-GFP, PVX-BAX, PVX-NIF1, PVX-flaA, and PVX-Vg_VWD were transformed into *A. tumefaciens* GV3101 for culturing, which then were centrifuged and resuspended with the buffer [10 mM 2-(N-morpholino) ethanesulfonic acid (MES), 10 mM MgCl_2_, and 100 μM acetosyringone] to OD_600_ = 0.6. After maintaining the suspension at room temperature and in the dark for 2 h, it was infiltrated into four to six leaves of *N. benthamiana* using sterile syringes. The symptoms were observed and photographed later.

For the callose deposition assay, the sampled *N. benthamiana* leaves were incubated in a mixed solution of acetic acid:glycerol:ethanol (1v:1v:3v) until the green color faded away, and then rinsed with 150 mM K_2_HPO_4_ for 30 min. Finally, samples were stained with aniline blue solution (150 mM K_2_HPO_4_, 0.05% w/v aniline blue) for 2 h, and the callose deposition was observed *via* fluorescence microscope using a DAPI filter (excitation filter 390 nm; dichroic mirror 420 nm; emission filter 460 nm) (Schenk and Schikora, 2015).

### 2.11. Phylogenetic analysis

A phylogenetic tree of *D. citri* for Vg_VWD and 11 other insect Vg-VWDs was constructed using the neighbor-joining (NJ) method with 1,000 bootstrap replicates in MEGA 7. Sequence information is provided in Supplementary Table 3.

### 2.12. Statistical analysis

All statistical analyses were performed using SPSS 24.0 software. A one-way ANOVA was used, followed by least-significant difference (LSD) multiple comparisons tests. Pairwise comparisons were performed by an independent samples *t*-test. Graphs were illustrated using GraphPad Prism 8.2.1 software. Data were expressed as means ± standard deviation.

## 3. Results

### 3.1. FlaA interaction with Vg_VWD

**Figure 1:**
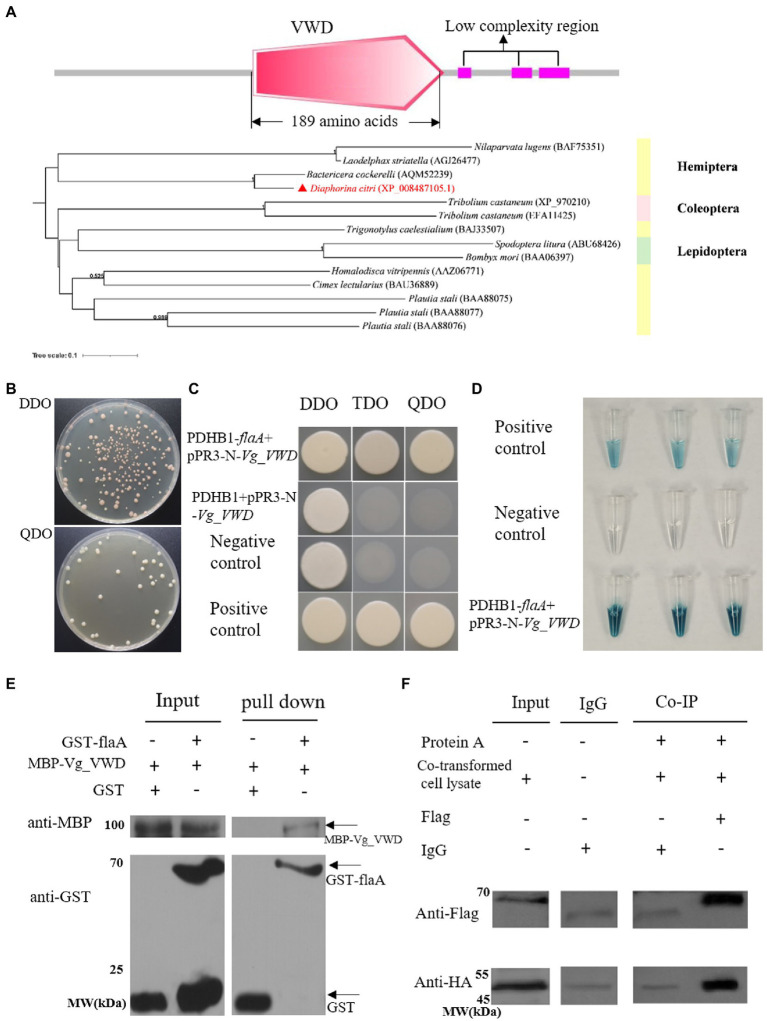
*C*Las flagellum (flaA) interacts with *D. citri* vitellogenin-like (Vg_VWD) and its molecular characterization. **(A)** The structural domain analysis of the Vg_VWD protein sequence and a phylogenetic tree containing Vg_VWD and other selected homologs from analysis by SMART software showed a VWD-conserved structural domain. **(B)** Yeast-two hybrid (Y2H) assay for the growth of yeast strain co-transformed with the bait and prey plasmids on DDO and QDO media. **(C)** The bait plasmid PDHB1-*flaA* and the prey-plasmid pPR3-N-*Vg_VWD* were co-transferred into NMY51 yeast, spotted on DDO, TDO, and QDO, and grow normally on selection medium TDO and QDO. PDHB1 + pPR3-N-*Vg_VWD* was verified for self-activation of the prey-plasmid and failed to grow on selection medium TDO and QDO. **(D)** Yeast cultures were blue in the *β*-galactosidase assay. The well-grown test yeasts were selected separately and subjected to overnight incubation in a DDO liquid medium, after which crude protein was extracted and the concentrations tested. Approximately 75 μL of crude protein was added to 5 μL of x-gal and incubated at 37°C for 2 h to observe the color change and photograph. **(E)** GST pull-down assay demonstrating the interaction of flaA with Vg_VWD. GST-flaA was the bait, and GST alone served as the control, with MBP-Vg_VWD serving as the prey. The bait protein or the GST control was incubated with MBP-Vg_VWD protein. Input and pull-down samples were probed with antibodies against GST or MBP for Western blot assays. **(F)** Co-IP detection of flaA interacting with Vg_VWD in Sf9 cells. Sf9 cells were transfected with the indicated plasmid combinations and the proteins were present in the lysate supernatant of Sf9 cells. The input group detected the target proteins in the lysate of co-transfected cells: Vg_VWD + Flag and HA + flaA with protein molecular weights of 65.1 kDa and 51.2 k Da, respectively. The IgG group excluded the heavy chain interference generated by protein A and IgG antibodies. The co-IP group was immunoprecipitated with Flag antibody (murine monoclonal antibody), IgG was used as control, and after WB analysis, the arrow marks the position of the target band.

To find *D. citri* proteins targeting *C*Las flagellum proteins, flaA was used as bait. The pDHB1-flaA plasmid passed the self-activation assay and functional validation (Supplementary Figure 1), and a Y2H screen was performed on the *D. citri* membrane library. As a result, 16 candidate proteins with potential interactions with flaA, of which 11 were annotated and five were unannotated (Supplementary Table 3). Following structural domain analysis and functional prediction, a vitellogenin-1-like protein XP_008487105.1 (named Vg_VWD thereafter) containing a conserved domain-VWD with 189 amino acids was selected for further analysis (Figure 1A). The phylogenetic analysis showed that the Vg-VWD sequences of *D. citri* and other representative Hemiptera insects clustered together. In addition, the Vg_VWD sequences of *D. citri* and the potato psyllid (*Bactericera cockerelli*) sequence were closest in the evolutionary relationship (Figure 1A).

The verification of the flaA and Vg_VWD cotransformants showed that the yeast grew well and formed a certain gradient on DDO and QDO (Figure 1B). The point-to-point Y2H method and *β*-galactosidase assays showed that flaA-Vg_VWD interacted in the yeast system (Figures 1C,D). Subsequent GST pull-down and Co-IP assays further confirmed the interaction between flaA and Vg_VWD (Figures 1E,F). Taken together, these results indicate that flaA interacts with Vg_VWD *in vivo* and *in vitro*.

### 3.2. *C*Las acquisition induces Vg_VWD upregulation in *Diaphorina citri*

**Figure 2:**
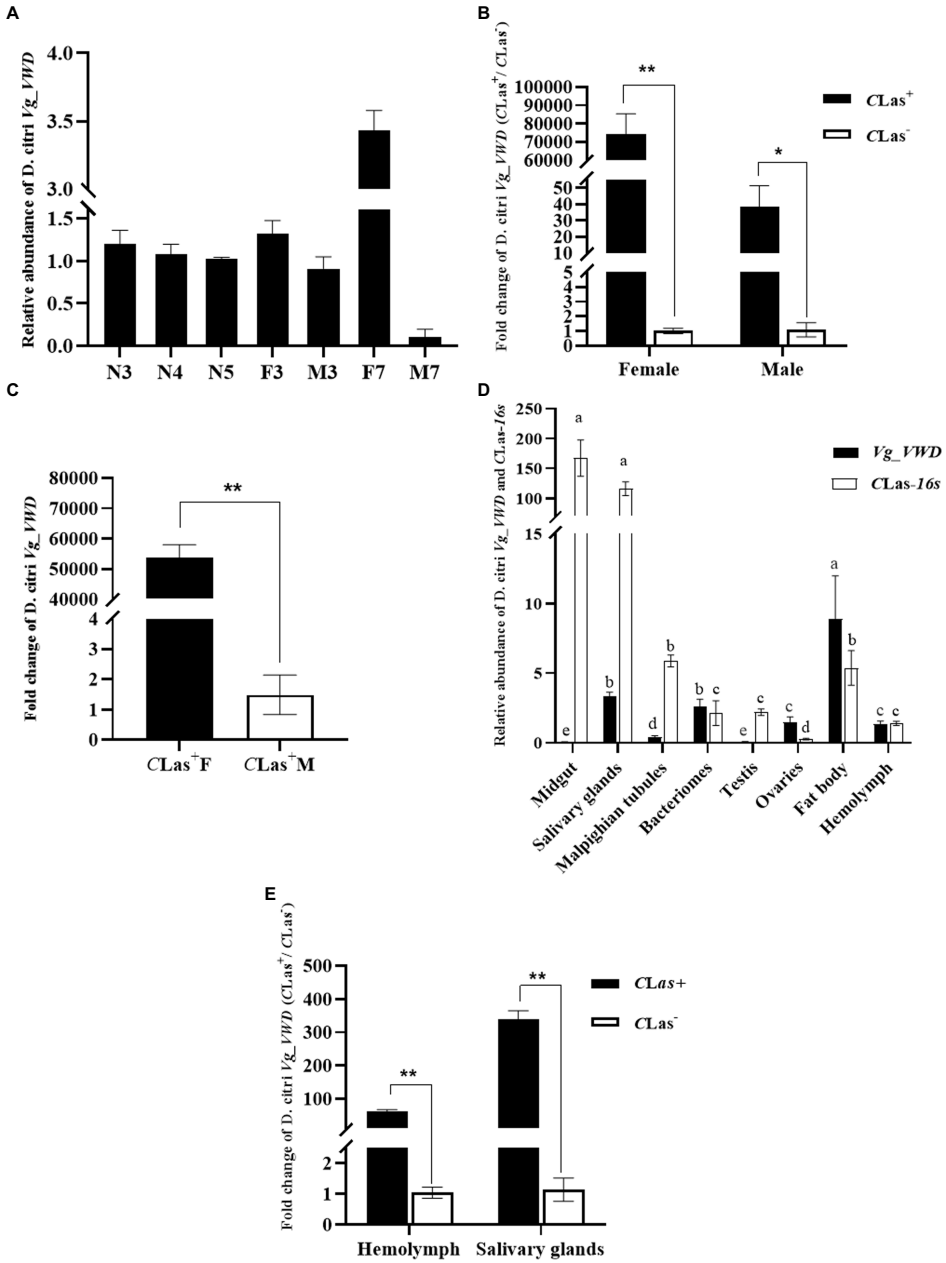
Expression profiles of *Vg_VWD* in different tissues of male and female individuals of *D. citri*, and sampled at different developmental stages. **(A)** Relative expression of *Vg_VWD* at different developmental stages (N3–N5, 3rd- to 5th instar nymphs; 3–7, 3, and 7 days after emergence as adults; F; female, M: male). **(B)** Relative expression of *Vg_VWD* between male and female of *C*Las-infected and uninfected *D. citri*. **(C)** Relative expression of *Vg_VWD* expression between male and female of *C*Las-infected *D. citri*. **(D)** Relative expression of *C*Las*-16 s* and *Vg_VWD* expression in different tissue of *C*Las-infected *D. citri* **(E)** Relative expression of *Vg_VWD* in the hemolymph and salivary glands of *C*Las-infected and uninfected *D. citri*. Data were analyzed using SPSS software, multiple comparisons using the one-way ANOVA followed by least significant difference (LSD) *post-hoc* tests at a *p*-value of 0.05. Pairwise comparisons were performed by independent samples *t*-test. The significant differences are indicated by * (*p* < 0.05) or ** (*p* < 0.01). Different letters in **(E)** indicate that the genes in the same middle are different in different types of samples (*p* < 0.05), and the gene expression is adjusted by log_10_ and then subjected to the one-way ANOVA. GraphPad software was used for drawing.

Quantitative RT-PCR was used to measure the transcript levels of the *Vg_VWD* gene in *D*. *citri*. In uninfected *D. citri*, the expression level of *Vg_VWD* gradually decreased in 3–5 instar nymphs, and Vg-VWD expression levels increased in females and decreased in males at 3–7 days after emergence (Figure 2A). The transcript abundance of *Vg_VWD* in *C*Las-infected *D. citri* was much higher than that in uninfected *D. citri* (*p* < 0.05), especially in females. This induction was 74,227-fold (*C*Las-infected/uninfected) in females, but only 38.3-fold (*C*Las-infected/uninfected) in males. In addition, the transcript abundance of *Vg_VWD* in *C*Las-infected females was 36,289-fold higher than that in *C*Las-infected males. These results indicate that *Vg_VWD* is expressed at significantly higher levels in females than males after infection (*p* < 0.05) (Figures 2B,C).

The proteomics analysis showed that *Vg* was upregulated in the hemolymph of the *C*Las-infected *D. citri* (Kruse et al., 2018). Here, we further analyzed the expression patterns of the *16srRNA* and *Vg_VWD* genes of *D. citri* in the midgut, salivary glands, malpighian tubules, bacteriomes, testes, ovaries, fat body, and hemolymph of the *C*Las-infected *D. citri* using RT-qPCR. The gene expression levels of 16S rRNA in the midgut and salivary gland were significantly higher than those in the other tissues (*p* < 0.05), while the transcription of *Vg_VWD* was highest in the fat body, followed by the salivary glands and bacteriomes (Figure 2D).

As insect hemolymph and salivary glands play important roles in the transmission of pathogens (Jiang et al., 2019; Chen et al., 2021), we next compared the transcription of *Vg_VWD* in the salivary glands and hemolymph of infected and uninfected *D. citri*. The results showed that the expression levels of *Vg_VWD* in the salivary gland and hemolymph were 338.39-fold and 61.67-fold higher in infected *D. citri* than those in uninfected *D. citri*, respectively (Figure 2E). Collectively, these results demonstrate that *C*Las acquisition induces *Vg_VWD* transcription in *D. citri*, with significant differences between males and females (*p* < 0.05).

### 3.3. Reducing Vg_VWD increased the titer of *C*Las in *Diaphorina citri*

**Figure 3:**
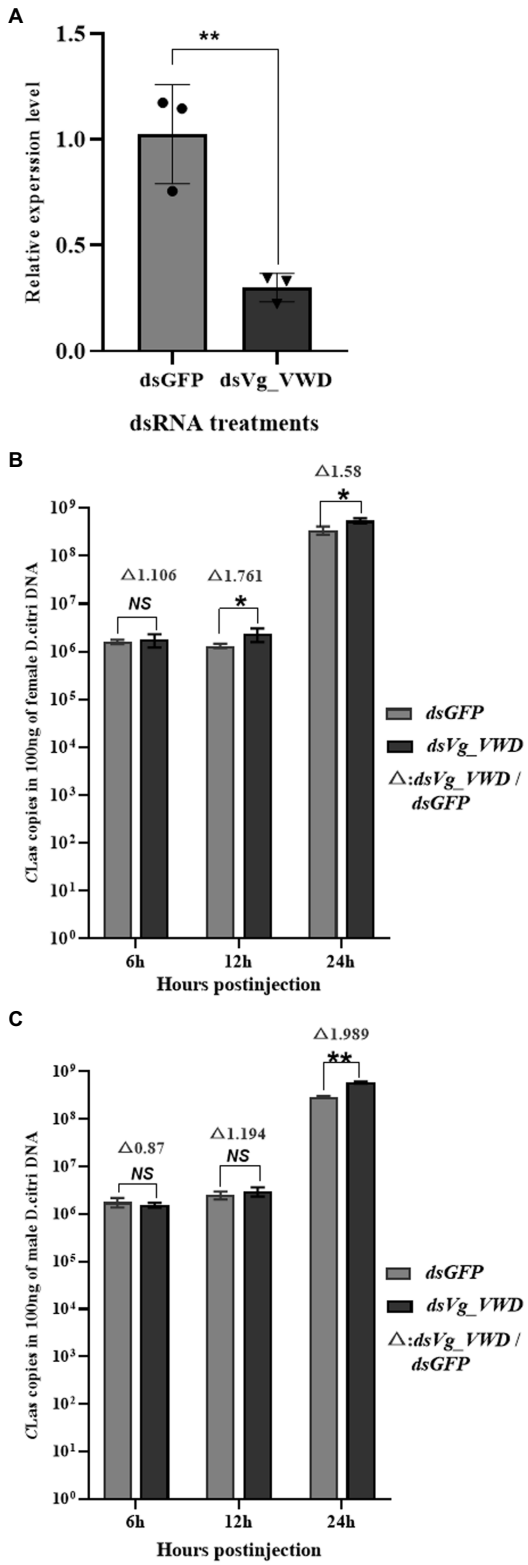
*D. citri Vg_VWD* silencing efficiency and detection of changes in *C*Las titer after silencing. **(A)** *Vg_VWD* silencing efficiency of female *D. citri*. **(B,C)** Are the detection of changes in *C*Las titer at 6, 12, and 24 h after the silencing, respectively. Data analysis was performed using SPSS software for independent samples *t*-test, the significant differences are indicated by * (*p* < 0.05) or ** (*p* < 0.01).

Next, according to the characteristics of *Vg_VWD* expression (Figure 2A), we constructed *Vg_VWD-*silenced *D. citri* using *dsVg_VWD-*mediated RNAi silencing to determine whether Vg_VWD is involved in *C*Las proliferation. After *dsVg_VWD* injection in *C*Las-infected *D. citri*, the expression of *Vg_VWD* was reduced by 70% compared with the same *dsGFP* injection dose for 24 h (Figure 3A). The *C*Las titer was detected in the *C*Las-infected male and female of *D. citri* at 6, 12, and 24 h after the injection. The results showed that the *C*Las titer of female *D. citri* was significantly different from the control group at 12 and 24 h (Figure 3B), with an increase of 1.76 and 1.58 times (*C*Las copies in 100 ng of female *D. citri* DNA), respectively. There was a significant difference in the *C*Las titer in male *D. citri* at 24 h compared with the control group, which was 1.98-fold higher (*C*Las copies in 100 ng of male *D. citri* DNA), whereas no significant differences were observed at 6 and 12 h (Figure 3C).

### 3.4. Vg_VWD suppresses BAX- and INF1-triggered hypersensitive cell death and inhibits flaA-induced callose deposition in *Nicotiana benthamiana*

**Figure 4:**
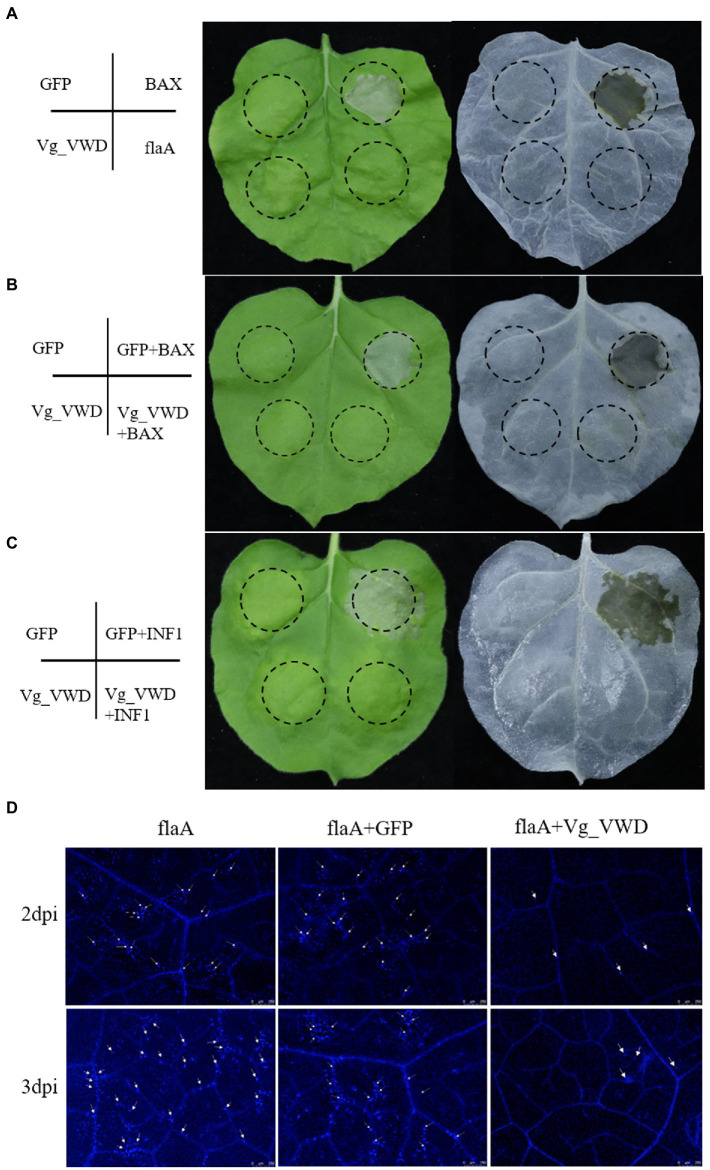
Transient expression of the Vg_VWD and flaA in *N. benthamiana*. **(A)** Symptoms on leaves with *GFP*, *BAX*, *Vg_VWD,* and *flaA* at 6 days postinoculation (dpi), where Vg_VWD and flaA postinoculation leaves showed no obvious symptoms. The *Agrobacterium* GV3101 (pJIC SA_Rep) strain harboring *GFP*, *Vg_VWD*, *flaA,* and *BAX*, resuspension with buffer, were infiltrated into the leaves of *N. benthamiana*. **(B,C)** The *Vg_VWD* suppressed the hypersensitive cell death triggered by *BAX* and *INF1* in *N. benthamiana* infiltrated with GV3101 carrying *GFP* and *Vg_VWD*, and followed 24 h later with *BAX* or *INF1* within the same regions (marked by the dashed circle). The leaves were harvested at 6 days after postinoculation of *BAX* and *INF1*, followed by photography and decolorization with ethanol. **(D)** Vg_VWD suppressed accumulation of callose caused by flaA. Infiltrated leaves with flaA after 2 and 3 days were sampled for Aniline-blue staining and photographed. Scale bars represent 250 μM. The experiment was repeated three times with at least 6 *N. benthamiana* leaves each time.

Since *Vg_VWD* was highly expressed in the salivary glands of the *C*Las-infected *D. citri* and directly interacts with *C*Las flagellin, we speculated that Vg_VWD may attach to the *C*Las flagellin protein during psyllid feeding along with the secretion of psyllid saliva into the citrus phloem, which is likely to be a stress factor. The mouse BAX and *Phytophthora infestans* INF1 are well-known inducers of cell death and are widely used to identify the PCD inhibitors of pathogens (Kamoun et al., 1998; Lacomme and Cruz, 1999). Vg_VWD was transiently expressed in *N. benthamiana* to evaluate its inhibitory effect on PCD, and the results showed that it could inhibit the BAX- and INF1-induced hypersensitivity response (Figures 4B,C). Callose deposition is one important indicator of plant immunity reaction, and the *C*Las flagellin has PAMP-triggered immunity and causes *N. benthamiana* leaf necrosis and callose deposition (Zou et al., 2012). Our results showed that Vg_VWD attenuates flaA-induced callose deposition in *N. benthamiana* (Figure 4D). Therefore, these results suggest that Vg_VWD can suppress *N. benthamiana* immunity and inhibit the phenomenon of callose deposition induced by flaA.

## 4. Discussion

Flagella are bacterial motor organs associated with tropism. Increasing numbers of studies have now shown that flagella play a central role in many bacterial infection processes, such as surface adhesion, biofilm formation, and the induction of host immunization (Duan et al., 2013; Chaban et al., 2015). *C*Las flagella genes have different expression patterns in different hosts, such as the flagella-like structures present in *C*Las cells isolated from *D. citri*, but they are not found in cells isolated from susceptible citrus (Andrade et al., 2020). Here, we found that the *C*Las flagellum (flaA) protein interacted with Vg_VWD in *D. citri*. This is one of the few reports of the protein–protein interaction between *C*Las and *D. citri* to our knowledge. Protein–protein interactions are an important way to understand the interactions between *C*Las and *D. citri*, but studies on which are lacking. Using protein interaction reporter technology, Ramsey et al. (2017) found that *D. citri* hemocyanin protein physically interacted with the *C*Las coenzyme A (CoA) biosynthesis enzyme phosphopantothenoylcysteine synthetase/decarboxylase, and *D. citri* myosin protein with the *C*Las pantothenate kinase. These interaction proteins may be able to provide important clues about the interaction relationship between *C*Las and *D. citri*.

**Figure 5:**
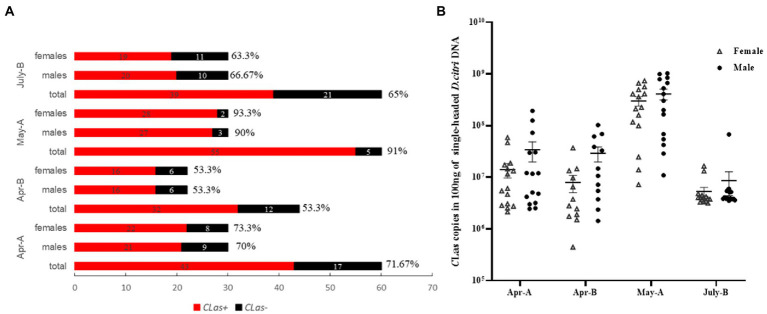
Detection of infection rate and titer of *C*Las in males and females of *D. citri* in the field. **(A)** Single male and single female were tested for *C*Las-infection rate in the field *D. citri*, and the percentages indicate the *C*Las-infection rates in each group. **(B)** *C*Las titers per 100 ng of DNA in males and females of the *C*Las-infection *D. citri*.

Over the past decade, insect Vg has been found to play important roles in reproductive development, immunity, and antioxidant activity (Park et al., 2018; Salmela and Sundström, 2018; Saleh et al., 2019). The Vg is usually synthesized and cleaved in the fat body of an insect (Dittmer et al., 2019; Wu et al., 2021). Vg, which also acts as a pattern recognition molecule to recognize pathogens, can interact with PAMPs such as bacterial outer membrane proteins, flagella, and pili, and acts as a PRR to induce host immunity (Liu et al., 2009; Arrese and Soulages, 2010). With the advancement of high-throughput sequencing and proteomic technologies, Vg has recently been discovered in insect salivary glands (Ji et al., 2013; Huang et al., 2018). Vg is also used as a saliva protein (Huang et al., 2021; Ji et al., 2021; Zeng et al., 2023). Transcriptome and hemolymph protein studies have revealed that Vg is upregulated in *C*Las-infected *D. citri* (Kruse et al., 2018; Jaiswal et al., 2021). This corroborates our detection of *Vg_VWD* expression in *C*Las-infected and uninfected *D. citri*. In addition, we found that *Vg_VWD* expression levels were significantly higher in females than in males, implying a sex bias. Reducing the expression of *Vg_VWD* in *D. citri* by RNAi interference led to a significant increase in *C*Las titers independent of insect sex, suggesting a general role in defense against *D. citri* infection. No significant difference was observed in the *C*Las infection rates between differentially *Vg_VWD*-expressed female and male *D. citri* collected in the field, which may indicate a neutral impact of *Vg_VWD* on the acquisition of *C*Las by *D. citri* (Figure 5A). Surprisingly, only a slightly higher titer of *C*Las in males than in females was detected here (Figure 5B) and in a previous study (Hosseinzadeh et al., 2019). *Vg_VWD* regulation-related immune pathways may play a balancing role between *C*Las and *D. citri* vector capacity, and therefore, we could also assume that the female is more susceptible to *C*Las than the male and thus maintains higher levels of Vg_VWD expression to counteract *C*Las. These viewpoints, however, require further validation.

*Vg_VWD* expression was confirmed by RT-qPCR to be highly abundant in the salivary glands and hemolymph of the *C*Las-infected *D. citri*, and the hemolymph detection result was consistent with that from a previous study (Kruse et al., 2018). We found that *Vg_VWD* was specifically highly expressed in the salivary glands of *C*Las-infection *D. citri*. In addition, it interacts with flaA, so we speculate that Vg_VWD may enter the plant host phloem by attaching to *C*Las in the absence of signal peptides and secretion. Therefore, we directly examined the influence of *Agrobacterium*-mediated transient expression of *Vg_VWD* in *N. benthamiana* as a substitute model plant for citrus. Transient expression showed that Vg_VWD inhibited the hypersensitivity reaction of *N. benthamiana* leaves caused by BAX and INF1, and also inhibited the phenomenon of leaf necrosis caused by BAX and INF1, confirming its function as a salivary protein. Recent studies have also reported that Vg in the salivary gland of the rice *planthopper* can inhibit plant immunity and the production of H_2_O_2_ during the feeding process as well as improve feeding fitness (Ji et al., 2021). FlaA has a conserved flg22 structural domain, which acts as a typical PAMP molecule and can induce plant immunity. In the present study, Vg_VWD was able to inhibit the plant immune response and suppress the phenomenon of callose deposition induced by flaA. From this perspective, Vg_VWD enters the plant by interacting with flaA, which may enhance the adaptation of *D. citri* feeding and create a favorable environment for *D. citri*. Conversely, both *C*Las and *C*Las flagellin can stimulate plant immunity (Zou et al., 2012; Ma et al., 2022). We speculate that the interaction of Vg_VWD with flaA may reduce the immune response induced by *C*Las invasion into plants, thus enhancing the early colonization of *C*Las in plants. The significance of this interaction is positive for both *D. citri* feeding and *C*Las early colonization, but negative for the plant. Meanwhile, the introduction of the *Vg_VWD* gene into a citrus genetic system could be used to study the relationship among *D. citri* feeding, *C*Las early colonization, and immune resistance of citrus in future, thereby providing new ideas for the prevention and control of Huanglongbing.

In summary, we present evidence that *C*Las–flaA interacts with *D. citri* Vg_VWD. As a regulatory factor, *Vg_VWD* was upregulated in *C*Las-infected *D. citri* compared with uninfected ones, and the *C*Las titer increased significantly after *Vg_VWD* was silenced. Furthermore, we also found that *Vg_VWD* was highly expressed in the salivary glands of the *C*Las-infected *D. citri*. In *N. benthamiana* plants, Vg_VWD inhibits plant immunity and plays a function similar to salivary proteins. This study not only deepens our understanding of the molecular interaction between *C*Las and *D. citri* but also provides a foundation for studying the roles that flaA and Vg_VWD may play together or separately in insect and plant hosts.

## Data availability statement

The datasets presented in this study can be found in online repositories. The names of the repository/repositories and accession number(s) can be found in the article/Supplementary material.

## Author contributions

TP, CZ, XW, and HY designed the experiments. TP and YY performed the experiments. CY participated in the rearing and collection of test insects. TP analyzed the data and wrote the manuscript. JH, XW, and CZ revised and embellished the manuscript. AH, SD, and LY provided the appropriate experimental apparatus and assistance. All authors contributed to the article and approved the submitted version.

## Funding

This study was supported by grants from the National Key Research and Development Program of China (2021YFD1400800), the National Natural Sciences Foundation of China (31871925 and 32160625), the Innovation Research 2035 Pilot Plan of Southwest University (SWU-5331000008), and the Science and Technology Project of Jiangxi Province (20225BCJ22005).

## Conflict of interest

The authors declare that the research was conducted in the absence of any commercial or financial relationships that could be construed as a potential conflict of interest.

## Publisher’s note

All claims expressed in this article are solely those of the authors and do not necessarily represent those of their affiliated organizations, or those of the publisher, the editors and the reviewers. Any product that may be evaluated in this article, or claim that may be made by its manufacturer, is not guaranteed or endorsed by the publisher.
