# Supplementary material for: Interaction between the flagellum of Candidatus Liberibacter asiaticus and the vitellogenin-like protein of Diaphorina citri significantly influences CLas titer
Source: Front Microbiol. 2023 Apr 18;14:1119619. doi: 10.3389/fmicb.2023.1119619 (PMC10152367; doi:10.3389/fmicb.2023.1119619)
Supplement: Supplementary file 3 [file Table_1.DOCX]

**Supplemental Table 1. The Primers used in this study**

| Primers(5’-3’ sequence) | sequence | Purpose |
| --- | --- | --- |
| FlaA-F | ATGACTAGTATTTTAACCAATCACT | full-length amplification |
| FlaA-R | TTAACCACGGAAAAGGGA |  |
| Vg-f | ATGGCTGTTGCCGCCACT | full-length amplification |
| Vg-r | TTAGTGATCACGTACCTTCTT |  |
| pBT3-N- flaA -F | gaattcctgcag**ggccattacggcc**ATGACTAGTATTTTAACCAATCACT | Construct pBT3-N- flaA plasmids |
| pBT3-N- flaA -R | acttaccatgg**ggccgaggcggcc**TTAACCACGGAAAAGGGA |  |
| pBT3-STE- flaA -F | ttttatgtaat**ggccattacggcc**ATGACTAGTATTTTAACCAATCACT | Construct pBT3- STE - flaA plasmids (Replace the stop codon with CC as required by the manufacturer) |
| pBT3-STE- flaA -R | ttcctgcagat**ggccgaggcggcc**CCACCACGGAAAAGGGA |  |
| pDHB1- flaA -F | cctaagaacgc**ggccattacggcc**ATGACTAGTATTTTAACCAATCACT | Construct pDHB1- flaA plasmids (Replace the stop codon with CC as required by the manufacturer) |
| pDHB1- flaA -F | ccccgacat**ggccgaggcggcc**CCACCACGGAAAAGGGA |  |
| pBT3-N-F | CAGAAGGATCCACCTTAC | Universal primers for pBT3-N plasmid sequencing |
| pBT3-N-R | AAGCGTGACATAACTAATTAC |  |
| pBT3-STE-F | TGGCATGCATGTGCTCTG | Universal primers for pBT3-STE plasmid sequencing |
| pBT3-STE-R | GTAAGGTGGACTCCTTCT |  |
| pDHB1-F | TTTCTGCACAATATTTCAAGC | Universal primers for pDHB1 plasmid sequencing |
| pDHB1-R | GTAAGGTGGACTCCTTCT |  |
| pPR3-N-F | GTCGAAAATTCAAGACAAGG | Universal primers for pPR3-N plasmid sequencing |
| pPR3-N-R | AAGCGTGACATAACTAATTAC |  |
| PPR3-N-vg-F | attaacaa**ggccattacggcc**ATGGCTGTTGCCGCCACT | Construct PPR3-N-vg plasmids |
| PPR3-N-vg-R | aactgatt**ggccgaggcggcc**TTAGTGATCACGTACCTTCTT |  |
| PEGX-4t-F | GGGCTGGCAAGCCACGTTTGGTG | Universal primers for PEGX-4t-1 plasmid sequencing |
| PEGX-4t-R | CCGGGAGCTGCATGTGTCAGAGG |  |
| PEGX-4t- flaA-F | gtggatccccg**gaattc**ATGACTAGTATTTTAACCAATCACT | Construct PEGX-4t- flaA plasmids |
| PEGX-4t- flaA-R | cgatgcggccg**ctcgag**TTAACCACGGAAAAGGGA |  |
| PEGX-4t- VWD-F | gtggatccccg**gaattc**ATGTACGCTGCATTCAAT | Construct PEGX-4t- VWD plasmids |
| PEGX-4t- VWD-R | cgatgcggccg**ctcgag**TTAGTTTCTTGGGGTGAT |  |
| Pvx- His-vg-F | caccagctagc**atcgat**ATGCACCATCACCATCACCATGCTGTTGCCGCCACT | Construct PGR-107- His-vg plasmids |
| PVX- vg-R | gcttatcggcg**gtcgac**TTAGTGATCACGTACCTTCTT |  |
| Pvx-His-flaA -F | caccagctagc**atcgat**ATGCACCATCACCATCACCATACTAGTATTTTAACCAATC | Construct PGR-107- His- flaA plasmids |
| PVX- flaA -R | gcttatcggcg**gtcgac**TTAACCACGGAAAAGGGATAAA |  |
| GAPDH-F | GACACTCACTCCTCCATCTTT | Quantative RT-PCR |
| GAPDH-R | GTATCCGTACTCGTTGTCATACC |  |
| OI1 | GCG CGTATG TAC GAG CGG CA | HLB detection |
| OI2c | GCC TCG CGA CTT CGC AAC CCA T |  |
| HLB4G | AGTCGAGCGCGTATGCGAA | *C*Las titer detection |
| HLBr | GCGTTATCCCGTAGAAAAAGGTAG |  |
| Vg_VWD-F | AACCACCCACAAGCTGAACA | Quantative RT-PCR |
| Vg_VWD -R | ACTTGGATTTTGGCGTTGGC |  |
| ds Vg_VWD -F | taatacgactcactataggg CAGAAAACAAATGGAACA | RNAI |
| ds Vg_VWD -R | taatacgactcactataggg AAGTCATGAGCACGTACC |  |
| dsGFP-F | taatacgactcactatagggCAGTTCTTGTTGAATTAGATG |  |
| dsGFP-R | taatacgactcactatagggTTTGGTTTGTCTCCCATGATG |  |

**Supplemental Table 2. Strains and plasmids used in this study**

| **Strains and plasmids** | **Characteristics** | **Source** |
| --- | --- | --- |
| **Strains** | | |
| Mach1-T1 | E.coli strain for plasmid construction | Shanghai Weidi Biotechnology Co.，Ltd |
| Escherichia coli BL21 | Prokaryotic expression strain |  |
| Roset（DE3） | Prokaryotic expression strain |  |
| NMY51 | Yeast strain |  |
| GV3101(PJIC SA_Rep) | Agrobacteria strain |  |
| SF9 cell | *Spodoptera frugiperda* cell | Zoonbio Biotechnology |
| **Plasmids** | | |
| pGEX-4t-1 | GST tag protein expression vector | Lab collection |
| pMAL-c2X | MBP tag protein expression vector |  |
| pBT3-STE | Vector for Y2H assay |  |
| pBT3-N | Vector for Y2H assay |  |
| pDHB_1_ | Vector for Y2H assay |  |
| pPR3-N | Vector for Y2H assay |  |
| PGR-107 | N. benthamiana transient expression |  |
| pFastBac1 | Co-IP protein expression vector |  |
