# Supplementary material for: Interaction between the flagellum of Candidatus Liberibacter asiaticus and the vitellogenin-like protein of Diaphorina citri significantly influences CLas titer
Source: Front Microbiol. 2023 Apr 18;14:1119619. doi: 10.3389/fmicb.2023.1119619 (PMC10152367; doi:10.3389/fmicb.2023.1119619)
Supplement: Supplementary file 4 [file Table_2.docx]

**Supplemental Table 2. Strains and plasmids used in this study**

| **Strains and plasmids** | **Characteristics** | **Source** |
| --- | --- | --- |
| **Strains** | | |
| Mach1-T1 | E.coli strain for plasmid construction | Shanghai Weidi Biotechnology Co.，Ltd |
| *Escherichia coli* BL21 | Prokaryotic expression strain |  |
| Roset（DE3） | Prokaryotic expression strain |  |
| NMY51 | Yeast strain |  |
| GV3101(PJIC SA_Rep) | *Agrobacteria* strain |  |
| SF9 cell | *Spodoptera frugiperda* cell | Zoonbio Biotechnology |
| **Plasmids** | | |
| pGEX-4t-1 | GST tag protein expression vector | Lab collection |
| pMAL-c2X | MBP tag protein expression vector |  |
| pBT3-STE | Vector for Y2H assay |  |
| pBT3-N | Vector for Y2H assay |  |
| pDHB_1_ | Vector for Y2H assay |  |
| pPR3-N | Vector for Y2H assay |  |
| PGR-107 | *N. benthamiana* transient expression |  |
| pFastBac1 | Co-IP protein expression vector |  |
