# Supplementary material for: Interaction between the flagellum of Candidatus Liberibacter asiaticus and the vitellogenin-like protein of Diaphorina citri significantly influences CLas titer
Source: Front Microbiol. 2023 Apr 18;14:1119619. doi: 10.3389/fmicb.2023.1119619 (PMC10152367; doi:10.3389/fmicb.2023.1119619)
Supplement: Supplementary file 5 [file Table_3.DOCX]

Table 3 Bait flaA candidate interacting proteins gene information

| **Protein** | **Name** | **Structural domain analysis** | **Function prediction** |
| --- | --- | --- | --- |
| XP_008477895.1 | *D.citri* 60S ribosomal protein L38 | Ribosomal_L38e  (PF01781) | Associated with ribosomal biological function. (Charoenkwan et al., 2021; Gopanenko et al., 2021) |
| XP_008468008.1 | *D*. *citri* 60S ribosomal protein L27 | KOW (SM000739)  Ribosomal_L27e  (PF01777) | Ribosomal proteins are associated with ribosome function (Wahl et al., 2002). |
| XP_017302266.1 | *D. citri* 40S ribosomal protein S7-like | Ribosomal_S7e  (PF01251) | Ribosomal proteins are associated with ribosome function. (Nomura et al., 2003). |
| XP_008477895.1 | *D. citri* 60S ribosomal protein L38 | Ribosomal_L38e  (PF01781) | Ribosomal proteins are associated with ribosome function. (Sanyal et al., 2000). |
| XP_008474491.1 | *D. citri* double-stranded RNA-specific editase B2-like | A_deamin  (PF02137) | Editase are enzymes that alter mRNA by catalyzing the site-selective deamination of adenosine residue into inosine residue(Maas et al., 1997; Seeburg et al., 1998). |
| XP_008487105.1 | *D.citri* vitellogenin-like | VWD  (SM000216) | It plays an important role in insect reproduction and immunity, and is able to bind to bacterial and viral particles. (Zhang et al., 2011; Sun et al., 2015; Park et al., 2018). |
| XP_026681929.1 | *D.citri* intraflagellar transport protein 57 homolog | IFT57  (PF10498) | An important component of intraflagellar transport (IFT) (Wei et al., 2013; Banerjee and Kane, 2017; Sorusch et al., 2019). |
| XP_026677571 | *D. citri* putative fatty acyl-CoA reductase CG8306 | NAD_binding_4  (PF07993)  Sterile (PF03015) | Has the c-terminal structural domain of fatty acyl coenzyme a reductase and can act as a fatty acyl coenzyme a reductase.  (Lienard et al., 2010; Teerawanichpan et al., 2010). |
| YP_009254299.1 | *D.citri* NADH dehydrogenase subunit 4 | Proton_antipo_M（PF00361） | Affects the production of ATP and reactive oxygen species. (Damm et al., 2012). |
| AVV28575 | *D.citri* cytochrome b | Cytochrome_B (PF00033) | Involved in electron transfer in the mitochondrial respiratory chain. (Gruschke et al., 2012; Seddigh et al., 2018) |
| XP_017299399 | *D.citri* V-type proton ATPase subunit S1-like | ATP-synt_S1  (PF05827) | v -ATPase hydrolyzes ATP to drive proton pumps and is involved in a variety of important intracellular and intercellular processes (Hiesinger et al., 2005). |
| XP_008486232.1 | *D.citri* uncharacterized | ATP-synt_S1  (PF05827) |  |
| XP_026680265 | *D.citri* uncharacterized | \ | \ |
| XP_026686374 | *D.citri* uncharacterized | \ | \ |
| LOC113466208 | *D.citri* uncharacterized | \ | \ |
| LOC113466505 | *D.citri* uncharacterized | \ | \ |

Note: Candidate proteins obtained by sequencing comparison of yeast spots grown on QDO.

Phylogenetic analysis

>D.citri_vg-VWD (189 aa)

YAAFNGQYNPVCVADGSSGQTFDNKTYPLNLEKDSWYVLMTSAPKQRKDNRVDYKTQRQE

NVTILVKQSGENKKELKIVLNNGEHVIDMQPSSSNNDGANAKIQVNKKDQRASKNSVTEV

TDNQNQRIAQIYALPSGEVIVNMPNHGLRLNYDGSRVQVQATDRFRDGVRGLCGSFTGEK

ATDFITPRN

>B.cockerelli_Vg-VWD (190 aa)

YYALNGQYTPVCVADGSSAQTFDNKTYPLNLEKDSWYVLMTSASKQRKNNNVDYKTQRQN

NVTILVKQSGDNKKDVQIILNNGEHVIEMQPASSSNNNNANAKIQVNKKDQRASKNSVTE

VTDSQNERIAQIYALPSEEVIVNIPSHGLSLNYDGSRVQVEANDRFRDGVRGLCGTFNGE

KATDFTTPRN

>L.striatella_Vg-VWD (194 aa)

SYNENYDYPTCSVSKNSISTFDNKTYSADLEGWHVMFASTPKNYNDNSGRYSASNSQSNS

FYKYKKVAILAKNSGSQRKAVKMLLGDNVIDITPSGSESNNNSPNANVQVNGNKMHIANN

RLASFEDFDGETLVEISVNDNGEVQVQSPSHGIAVNHDGANFMIDADSYYRGEVRGLCGT

YSGDKYTDFTTPKK

>C.lectularius_Vg-VWD (177 aa)

QTYFRAQQFPTCVVDKNLATTFDNKSFPVKLGDCWHVLAHSLGVFTHDPEDKYFGALVRE

HESDKKELVLVFGENVVEVKPTSSSDKVGVVKVNGQTAEFTQTKVAKFEDNFGYTFFQVY

ALPTGAVRMYSPLAGVEVVYDGARVKLQVSNTFRGQLRGLCGTFNGEDVDDFTCPNN

>H.vitripennis_Vg-VWD (184 aa)

NHIFKGQQFPTCVVDNNWAQTFDNKSYPIKLGKCWHAMFHYTPKEDPTSSESTNDYDEDE

ISILVQEASSSNEKELMIVLGGYNIYMQPTPGNSPAQVTVNGQQTPVSKSYLTELFDQNG

NTLAQMYARPNGEVHFYAAQQDIKVQYDGTAVKVKAQNSYRSETRGLCGTFNTQPVDDFT

TPQG

>P.stali_Vg1-VWD (184 aa)

DYFDTKNYATCGVDGNGVVTTFNGQSYSIDYEDFTYVLVYALPGEHFESSEDSSEEYEST

YLGGFSVVAKDYGSNQKEGRILLDDDKIEMRPSGNGVAVTANGEMVQVEEKKITTWENGN

NKFEVFAMPGGGNAAFFFPNHGLEVYYDGKRLVVSVSNHHRDRVRGICGTMDGEPSYDFT

TPAN

>P.stali_Vg2-VWD (185 aa)

YYSTPYATCAIDNTEASTFNNRTYPIELGNCFHVLAMSIPNKYQQQSQESSEYSFNQDDI

VIFAREPTPQKKEVQVIFGYDVVSFEPTGASDVTVKVNGESIPISASKIARWENGPYELQ

VYALPGKNNVVAYFNNHDFQADSFQIYFDGHRAAITMPNSYRNKLRGLCGTFDGEPFNDF

TTSAN

>P.stali_Vg3-VWD (186 aa)

QQVFNRNPAGTCTLDAVNTTTFNNKTYPVNLGDCYHVVAMYAPPTSQEGQGGQQYSEGTG

QFQRGFAILVKQTDSHKKVVKVVVGNEAVTLEPAGPGGVSVTYNGAAIPLSEHAVYRWEA

GPHRVQAYVQPGTTNVVLQFTNHSIQVVHDGQRVQIQIPNSYRNMVRGLCGTFDGEPVND

YTSPRN

>T.caelestialium_Vg-VWD (180 aa)

NYLYQGQGYPTCSIDKNQATTFDNKSYPLNLDQSWTLMANFIPKEKFDYSASSPRSESIV

VIGVRQTGSDKKEVRMLLGYDQVELLPEGKTGVVKFNGRKANFDTQTPDYFKDSNGQIVV

QVLALSDNTVRIVSQKYNIQMLYDGQRVQLQASNAYRGKMYGLCGNFDGEAINDFTSPKN

>T.castaneum_Vg1-VWD (193 aa)

QALKYDTYRPICVVDKTQTSTWDNKTYPSSFSNGWTVLLHYVPRRPSSSQNKPYESVQEQ

LNELVESYIVYARASEQSHSQKEIQIVLQMPCTNGKVVKIAMKPSSKGPKVLIDEQEVKY

DTEHASDAYDGAIQIYGLPNQEVKLEIRDAFYAIFNGQTLKLTATNSKFRDASRGLCGTF

TGEQETDFLGPDN

>T.castaneum_Vg2-VWD (193 aa)

GYALNYETYKANCMVDSCAVNTLDNRTFPIDLSKDWVVLLHYVPRRPSPIKNQPYLTVPE

QLNQQVEGYIVYGRSYGDAKKEIKMVIQSPDTLSKVIDISLKPSESSSYPRLFVQGQEIK

YDDQGSHMYDGYMQAYRLPNQETKVQVYNAFSVIYDGVRAKIEITNDKFRDAARGLCGTF

TNEQETDFTHPGN

>B.mori_Vg-VWD (179 aa)

NYFTGHQYQPYCSIDGTRIHTFSNRSYEYPLSRSWHVVMQDESTQRGNWHELAILSRRQQ

RDQQEIYISYKSESGQDLEIEIQPASGDSAYQVKVTTNTKKITDDDLTMYWDDVKEQPFL

QYHTHKDGVLVINIEDDRIRAIYDGQRFVVFTQDYRNSTRGICGRMSGEQRDDYLTPEG

>N.lugens_Vg-VWD (186 aa)

TTITQHVLSARTPSAHSTTRPTHADLEGWHVMFASTPKNFNDNSGRYSASNSQSNSFYKY

KKVVVLAKNAGSQRKAVKMLLGENVIDINPSGSESSDNSPNANVQVNGNKVQIANNRMAS

FDDFDGETLVEISVTDNGEVQVQSSSHGIAVYHDGANFIIDADSYHRGEVRGLCGTYSGD

KYSPLH

>S.litura_Vg-VWD (179 aa)

NYAGNGQYQPFCTIDDNKVKTFGNRSINYELSRSWHLVMQEESNENRGQWNEMVILARRP

SQQEQELYISYITETGKDLEIEIKPSQSKRPNVQVKTNSKKISEGDLTVYWDDVQDEPLL

EYFTEADDVLMLNIRDGRLRAMYDGQRLVLTTQDHRKTNRGICGQNSGEARDDYQTPAG

**REFERENCES**

Banerjee, S., and Kane, P. M. (2017). Direct interaction of the Golgi V-ATPase a-subunit isoform with PI(4)P drives localization of Golgi V-ATPases in yeast. *Mol. Biol. Cell.* 28, 2518-2530. doi: 10.1091/mbc.E17-05-0316

Charoenkwan, P., Chotpatiwetchkul, W., Lee, V. S., Nantasenamat, C., and Shoombuatong, W. (2021). A novel sequence-based predictor for identifying and characterizing thermophilic proteins using estimated propensity scores of dipeptides. *Sci. Rep.* 11, 1-15. doi: 10.1038/s41598-021-03293-w

Damm, F., Bunke, T., Thol, F., Markus, B., Wagner, K., Goehring, G., et al. (2012). Prognostic implications and molecular associations of NADH dehydrogenase subunit 4 (ND4) mutations in acute myeloid leukemia. *Leukemia.* 26, 289-295. doi: 10.1038/leu.2011.200

Gopanenko, A. V., Kolobova, A. V., Meschaninova, M. I., Venyaminova, A. G., Tupikin, A. E., Kabilov, M. R., et al. (2021). Knockdown of the mRNA encoding the ribosomal protein eL38 in mammalian cells causes a substantial reorganization of genomic transcription. *Biochimie.* 184, 132-142. doi:10.1016/j.biochi.2021.02.017

Gruschke, S., Rompler, K., Hildenbeutel, M., Kehrein, K., Kuehl, I., Bonnefoy, N., et al. (2012). The Cbp3-Cbp6 complex coordinates cytochrome b synthesis with bc(1) complex assembly in yeast mitochondria. *J. Cell Biol.* 199, 137-150. doi: 10.1083/jcb.201206040

Hiesinger, P. R., Fayyazuddin, A., Mehta, S. Q., Rosenmund, T., Schulze, K. L., Zhai, R. G., et al. (2005). The v-ATPase V-0 subunit a1 is required for a late step in synaptic vesicle exocytosis in Drosophila. *Cell.* 121, 607-620. doi: 10.1016/j.cell.2005.03.012

Lienard, M. A., Hagstrom, A. K., Lassance, J.-M., and Lofstedt, C. (2010). Evolution of multicomponent pheromone signals in small ermine moths involves a single fatty-acyl reductase gene. *Proc. Natl. Acad. Sci. U.S.A.* 107, 10955-10960. doi: 10.1073/pnas.1000823107

Maas, S., Melcher, T., and Seeburg, P. H. (1997). Mammalian RNA-dependent deaminases and edited mRNAs. *Curr. Opin. Cell Biol.* 9, 343-349. doi: 10.1016/s0955-0674(97)80006-3

Nomura, T., Mochizuki, R., Dabbs, E. R., Shimizu, Y., Ueda, T., Hachimori, A., et al. (2003). A point mutation in ribosomal protein L7/L12 reduces its ability to form a compact dimer structure and to assemble into the GTPase center. *Biochemistry.* 42, 4691-4698. doi: 10.1021/bi027087g

Park, H. G., Lee, K. S., Kim, B. Y., Yoon, H. J., Choi, Y. S., Lee, K. Y., et al. (2018). Honeybee (Apis cerana) vitellogenin acts as an antimicrobial and antioxidant agent in the body and venom. *Dev. Comp. Immunol.* 85, 51-60. doi: 10.1016/j.dci.2018.04.001

Sanyal, S. C., and Liljas, A. (2000). The end of the beginning: structural studies of ribosomal proteins. *Curr. Opin. Struct. Biol.* 10, 633-636. doi: 10.1016/s0959-440x(00)00143-3

Seddigh, S., and Darabi, M. (2018). Functional, structural, and phylogenetic analysis of mitochondrial cytochrome b (cytb) in insects. *Mitochondrial DNA Part A.* 29, 236-249. doi: 10.1080/24701394.2016.1275596

Seeburg, P. H., Higuchi, M., and Sprengel, R. (1998). RNA editing of brain glutamate receptor channels: mechanism and physiology. *Brain Res. Rev.* 26, 217-229. doi: 10.1016/s0165-0173(97)00062-3

Sorusch, N., Yildirim, A., Knapp, B., Janson, J., Fleck, W., Scharf, C., et al. (2019). SANS (USH1G) Molecularly Links the Human Usher Syndrome Protein Network to the Intraflagellar Transport Module by Direct Binding to IFT-B Proteins. *Front. Cell Dev. Biol.* 7, doi: 10.3389/fcell.2019.00216

Sun, C., and Zhang, S. (2015). Immune-Relevant and Antioxidant Activities of Vitellogenin and Yolk Proteins in Fish. *Nutrients.* 7, 8818-8829. doi: 10.3390/nu7105432

Teerawanichpan, P., Robertson, A. J., and Qiu, X. (2010). A fatty acyl-CoA reductase highly expressed in the head of honey bee (*Apis mellifera*) involves biosynthesis of a wide range of aliphatic fatty alcohols. *Insect Biochem. Mol. Biol.* 40, 641-649. doi: 10.1016/j.ibmb.2010.06.004

Wahl, M. C., and Moller, W. (2002). Structure and function of the acidic ribosomal stalk proteins (vol 3, pg 99, 2002). *Curr. Protein Pept. Sci.* 3, doi: 10.2174/1389203023380756

Wei, Q., Xu, Q., Zhang, Y., Li, Y., Zhang, Q., Hu, Z., et al. (2013). Transition fibre protein FBF1 is required for the ciliary entry of assembled intraflagellar transport complexes. *Nat. Commun.* 4, doi: 10.1038/ncomms3750

Zhang, S., Wang, S., Li, H., and Li, L. (2011). Vitellogenin, a multivalent sensor and an antimicrobial effector. *Int. J. Biochem. Cell Biol.* 43, 303-305. doi: 10.1016/j.biocel.2010.11.003
